# *In vitro* Inhibition of HIV-1 by Cyclotide-Enriched Extracts of *Viola tricolor*

**DOI:** 10.3389/fphar.2022.888961

**Published:** 2022-05-27

**Authors:** Carina Conzelmann, Edin Muratspahić, Nataša Tomašević, Jan Münch, Christian W. Gruber

**Affiliations:** ^1^ Institute of Molecular Virology, Ulm University Medical Center, Ulm, Germany; ^2^ Center for Physiology and Pharmacology, Medical University of Vienna, Vienna, Austria

**Keywords:** plant extracts, *Viola tricolor*, anti-HIV, antiviral, cyclotides, cysteine-rich peptides

## Abstract

Since viral infectious diseases continue to be a global health threat, new antiviral drugs are urgently needed. A unique class of therapeutic compounds are antimicrobial peptides (AMPs). They can be found in humans, bacteria and plants. Plants express a wide variety of such defense peptides as part of their innate immune system to protect from invading pathogens. Cyclotides are non-classical AMPs that share a similar structure. Their unique topology consists of a circular peptide backbone and disulfide bonds. In previous studies they have been attributed to a wide range of biological activities. To identify novel cyclotides with antiviral activity, we established a library of plant extracts largely consisting of cyclotide-rich species and screened them as inhibitors of HIV-1 infection. Subsequent extraction and fractionation revealed four cyclotide-containing subfractions from *Viola tricolor* with antiviral activity. These subfractions inhibited HIV-1 infection with IC_50_ values between 0.6 and 11.2 μg/ml, and selectivity indices of up to 8.1. The identification and characterization of antiviral cyclotides and the determination of the antiviral mechanisms may allow to develop novel agents to combat viral infections. Therefore, cyclotides represent a natural source of bioactive molecules with prospects for development as therapeutics.

## Introduction

Viral infectious diseases continue to be a global threat to human health (WHO, 2020), as best illustrated by the ongoing SARS-CoV-2 pandemic. The development of preventive or therapeutic antiviral drugs allowed to effectively treat several viral diseases, but most approved antivirals target only a limited number of viral pathogens such as HIV-1, HCV, or herpes viruses. In comparison, the arsenal of drugs available to combat the more than 200 known human pathogenic viruses is very limited with no specific antiviral therapies against most of them (Nii-Trebi, 2017; WHO, 2022). Therefore, new drugs are needed, especially those that are less prone to resistance induction (Mahlapuu et al., 2016; Browne et al., 2020) and that have a broad activity which prepares for reemerging or emerging pandemic viruses (Kuroki et al., 2021). One unique class of therapeutic compounds are peptides which attained much attention over the course of the 20th century (Lau and Dunn, 2018). Peptides are recognized for their high selectivity and efficacy and are relatively safe and well tolerated (Mahlapuu et al., 2016; Wang et al., 2022). Peptide drugs now account for ∼7% of the total number of approved pharmaceuticals (Al Musaimi et al., 2021) and this number is expected to increase in the near future because of the tremendous progresses made in peptide synthesis and purification (Wang et al., 2022).

**FIGURE 1:**
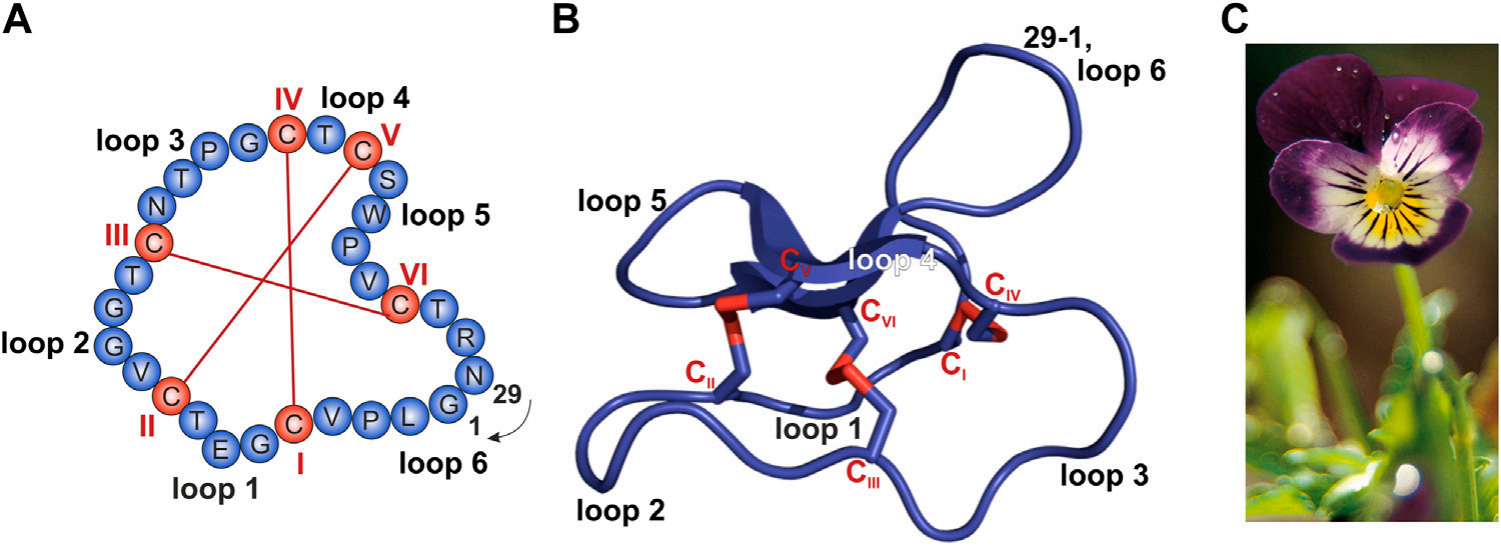
Sequence and structure of the prototypic cyclotide kalata B1 and photograph of *Viola tricolor*. **(A,B)** The cyclized peptide kalata B1 (PDB 1NB1) comprises 29 residues (blue) with six cysteine residues (C; red; Roman numerals) which form three disulfide bonds establishing the typical cyclic cystine knot (CKK) motif. The cyclization point between residue 1 and 29 is indicated. **(A)** Amino acid sequence obtained from the CyBase databank (Wang et al., 2007). **(B)** Cartoon of prototypical cyclotide structure of kalata B1 prepared with PyMol. **(C)** Photograph of *Viola tricolor*, kindly provided by *C. Gründemann*, courtesy of Weleda AG, Schwäbisch-Gmünd, Germany.

Antimicrobial peptides (AMPs) can also be found in humans, bacteria and plants (Huan et al., 2020). Plants have developed mechanisms to protect themselves against invading pathogens such as viruses, bacteria, fungi, nematodes and insects (Mott et al., 2014; Nishad et al., 2020). Since they only have the innate immune response, a wide variety of peptides is expressed (Mott et al., 2014; Mammari et al., 2021), many of which are rich in cysteines. Plant cysteine-rich peptides have been isolated from thousands of species (Liu et al., 2017); they are extremely diverse with hundreds of different peptides produced by a single plant species. These peptides are classified into different families mainly based on their cysteine spacing, disulfide bond network and three-dimensional fold (Silverstein et al., 2007; Tam et al., 2015; Hellinger and Gruber, 2019; Li et al., 2021). One of these families are cyclotides, which are circular, disulfide-rich peptides and typically consist of 28–37 amino acids (de Veer et al., 2019). They comprise a typical structural architecture, termed the cyclic cystine knot (CKK) motif. This is characterized by a head-to-tail cyclized peptide backbone and interlocking arrangement of three disulfide bonds (Figure 1). Due to this unique topology, they exhibit exceptional resistance to thermal, chemical and enzymatic degradation (Colgrave and Craik, 2004). Cyclotides are non-classical AMPs since they do not exhibit the typical AMP structure comprising a cationic amphipathic α-helical or β-sheet structure (Mahlapuu et al., 2016).

Cyclotides do not occur in all plants, but were isolated from flowering plants—including many medicinal plants with ethnopharmacological relevance—belonging to Violaceae, Rubiaceae, Cucurbitaceae, Fabaceae, and Solanaceae (Gruber et al., 2008; Poth et al., 2011, 2012; Nguyen et al., 2013; Burman et al., 2015; de Veer et al., 2019). Typically, cyclotides do not appear as singletons, but a single plant produces dozens of cyclotides (Hellinger et al., 2015; de Veer et al., 2019). Most plants have their own individual suite of cyclotides and most cyclotides are unique to a particular plant meaning that it is uncommon for one cyclotide to occur in multiple plant species (Gruber et al., 2008). However, variations in cyclotide content between tissues as well as over different seasons or in different geographic locations have been reported (de Veer et al., 2019). For example, in *Viola tricolor* (Figure 1C), a member of the Violaceae family, we previously isolated and characterized 164 different cyclotides (Hellinger et al., 2015). This plant is a common horticultural plant, and has been used in traditional medicine for heat-clearing, detoxification, and relieving coughs (Tang et al., 2010). *Viola tricolor* is also widely used in Russian official medicines, and a monograph on this plant is even included in the Russian Pharmacopoeia (Shikov et al., 2014, 2017, 2021). Since violaceous plants at a global scale may be the source to as many as 150,000 individual cyclotides, this commercially available medicinal herb may be a suitable starting point for bioactivity-guided screening studies (Hellinger et al., 2015). As a part of the plant host defense, cyclotides were shown to be insecticidal (Jennings et al., 2001), hemolytic (Wang et al., 2008), anti-bacterial (Strömstedt et al., 2017; Slazak et al., 2018), anti-fungal (Slazak et al., 2021), anti-cancer (Pinto et al., 2018), anti-fouling (Göransson et al., 2004), and antiviral (Daly et al., 2004; Ireland et al., 2008; Wang et al., 2008; Gerlach et al., 2013). It is hypothesized that the diversity of cyclotides in a single plant may reflect a strategy to overcome resistance development by pathogens or pests (de Veer et al., 2019).

Therefore, cyclotides represent a rich natural source of bioactive molecules with prospects for development as therapeutics against cancer and infectious diseases (Mammari et al., 2021). Making use of the natural evolution, a huge variety of already optimized peptides can be assessed (Lazzaro et al., 2020; Atanasov et al., 2021). Peptides have a good efficacy compared to small molecules (Wang et al., 2022) and a good safety profile because their degradation products are natural amino acids and they are generally less immunogenic than recombinant proteins or antibodies (Mahlapuu et al., 2016). In contrary, first-generation antivirals have severe side effects due to pour specificity (Mammari et al., 2021). Cyclotides are expected to be broadly active due to their direct mode of action destabilizing lipid membranes (Henriques and Craik, 2012; Henriques et al., 2012) which will most likely prevent resistance development (Mahlapuu et al., 2016; Browne et al., 2020; Huan et al., 2020). Finally, peptides can be chemically modified and structure-activity-relationship studies can help to improve therapeutic features such as bioavailability, specificity and bioactivity (Browne et al., 2020; Huan et al., 2020; Dijksteel et al., 2021; Wang et al., 2022). Typical AMPs have a low oral and systemic bioavailability due to enzymatic degradation (Mahlapuu et al., 2016), but cyclotides have an exceptional resistance to degradation because of their unique topology (Colgrave and Craik, 2004), making them a promising class of antivirals (Lau and Dunn, 2018). And indeed, anti-HIV activity was shown for the prototypic cyclotide kalata B1, but also other cyclotides like kalata B8, varv E and circulins A and B (Gustafson et al., 1994; Daly et al., 2004, 2006; Wang et al., 2008). Several classical AMPs are already on the market and further AMPs are currently evaluated in clinical trials (Mahlapuu et al., 2016). For example, the antiviral peptide T20 (enfuvirtide) is FDA-approved and commercially available (Huan et al., 2020). Other plant-derived peptides are also in clinical development. For example, griffithsin, a lectin isolated from the red alga *Griffithsia sp*., is investigated in clinical studies (NCT02875119; NCT04032717) as an anti-HIV microbicide for prevention of sexual transmission (Lee, 2019). Since it recognizes mannose, it has a broad-spectrum antiviral activity and was shown to inhibit SARS-CoV-2 recently (Cai et al., 2020). Additionally, the first cyclotide (called T20K) is currently in clinical trials for multiple sclerosis (Gründemann et al., 2019).

As nature-derived products are attractive candidates for translational application (Atanasov et al., 2021), we aimed to identify novel antiviral cyclotides. To this end, we established a library of plant extracts largely consisting of species with ethnopharmacological relevance that have previously been identified as a rich source of cyclotides or cyclotide-like knottin peptides. This encompasses ten plant species: *Viola odorata*, *Viola tricolor*, *Psychotria solitudinum*, *Palicourea tomentosa*, *Carapichea ipecacuanha, Bryonia alba*, *Beta vulgaris*, *Sambucus nigra* as well as *Momordica charantia* (He et al., 2013; Koehbach et al., 2013; Karpyuk et al., 2015; Fahradpour et al., 2017; Álvarez et al., 2018; de Veer et al., 2019; Retzl et al., 2020). Using solvent and solid-phase extraction yielded the plant extracts enriched in peptides. These extracts were analyzed for cytotoxic and anti-HIV-1 activity in cell culture-based infection assays. The resulting inhibitory concentration (IC_50_) and cell toxic concentration (CC_50_) were used to calculate selectivity indices and select the plants species for more detailed analysis. Using these criteria, the particular extract from *Viola tricolor*—a species used in traditional medicine and listed in the European Pharmacopoeia—had the most selective antiviral activity and was analyzed more thoroughly. To confirm the presence of cyclotides as bioactive molecule class in the extract, we applied (sub) fractionation by HPLC and mass spectrometry and identified several mixtures of cyclotides. These mixtures of cyclotides were subsequently analyzed again for IC_50_, CC_50_ and selectivity indices. Lastly, in an approach to determine the antiviral mechanism, we performed pre-treatment experiments of the virions with the cyclotides.

## 2 Materials and Methods

### Plant Material

Dried plant material of *Viola odorata* L. (total herb, batch no. 26055; origin: Serbia), *Viola tricolor* L. (total herb, batch no. 28645; origin: Bulgara), *Bryonia alba* L. (root, batch no. 24769; origin: Albania), *Beta vulgaris* L. (beet, batch no. 33861; origin: Uzbekistan), *Sambucus nigra* L. (flowers, batch no. 30087; origin: Hungary; and leaves, batch no. 13889; origin: Serbia), *Carapichea ipecacuanha* (Brot.) L. Andersson (root, batch no. 14553; origin: Costa Rica) and *Momordica charantia* L. (fruit, batch no. 28714; origin: India) was purchased from Alfred Galke GmbH (Bad Grund, Germany). The samples of *Psychotria solitudinum* Standl. (leaves) and *Palicourea tomentosa* (Aubl.) Borhidi (formerly known as *Psychotria poeppigiana*) (leaves) were collected in La Gamba (Costa Rica) (Koehbach et al., 2013). Dry material was stored at room temperature in the dark and was powdered just before extraction.

### Plant Extraction

The plant extracts and peptide-enriched fractions were prepared using previously established protocols (Muratspahić et al., 2021). Briefly, the peptides were extracted from dried plant material (50 g) using a mixture of methanol/dichloromethane, 1:1 (v/v), overnight and under continuous agitation at room temperature. Following removal of the plant material and filtration with 0.5 volume of ddH_2_O, the peptide-containing methanol/water phase was separated from the organic phase. The aqueous phase was subsequently concentrated using a rotary evaporator, lyophilized, and subjected to C_18_ solid-phase extraction. The dried, crude extract was dissolved in 5% acetonitrile/95% ddH_2_O (v/v) and loaded onto the C_18_ material ZEOprep 60 Å, irregular 40–64 µm (Zeochem, Uetikon, Switzerland). The column was equilibrated with solvent A (99.9% ddH_2_O/0.1% trifluoroacetic acid, v/v) and washed with 10–30% solvent B (90% acetonitrile/9.9% ddH_2_O/0.1% trifluoroacetic acid, v/v/v). The peptide-containing fractions were separated from hydrophilic components by elution with 50–80% solvent B, depending on the plant species. A mass fingerprint of peptide-enriched fractions was determined by MALDI-TOF mass spectrometry (MS) using a MALDI-TOF/TOF 4800 analyzer (AB Sciex, Framingham, MA, United States) in a reflector positive ion mode with 2,000 to 10,000 total shots per spectrum and a laser intensity of 3,500. 0.5 µl of each sample was mixed with 3 µl of a matrix solution and spotted directly onto the MALDI target plate. An α-cyano-hydroxyl-cinnamic acid (α-CHCA) matrix (Sigma–Aldrich, St. Louis, MO, United States), dissolved in ddH_2_O/acetonitrile/trifluoroacetic acid, 50/49.9/0.1% (v/v/v) with a final concentration of 5 mg/ml was used. Spectra were acquired, processed, and analyzed using the Data Explorer Software (AB Sciex). Most abundant peptides of extracts were labelled by molecular weight and, if applicable, identified by comparison to databases, such as Cybase (www.cybase.org.au; Wang et al., 2007) or previously published literature (Hellinger et al., 2015).

### Bioactivity-Guided Fractionation of *Viola tricolor*

The bioactivity-guided fractionation of the cyclotide-enriched *V. tricolor* extract was carried as previously described (Hellinger et al., 2014; Muratspahić et al., 2021). The extract was dissolved in 5% solvent B and manually loaded onto the preparative Phenomenex Jupiter C_18_ column (250 mm × 21.2 mm, 10 μm, 300 Å; Phenomenex, Aschaffenburg, Germany). Following preparative fractionation, a fractionation round 2 of *V. tricolor* was performed on a semipreparative Kromasil C_18_ column (250 × 10 mm, 5 μm, 100 Å). The mobile phase consisted of solvent A (99.9% ddH_2_O/0.1% trifluoroacetic acid, v/v) and solvent B (90% acetonitrile/9.9% ddH_2_O/0.1% trifluoroacetic acid, v/v/v). The preparative RP-HPLC fractions were collected automatically on a Dionex 3000 LC unit (Dionex, Amsterdam, Netherlands) machine using a linear gradient of solvent B between 5% and 65% at a flow rate of 8 ml/min while the semipreparative RP-HPLC were collected manually with a linear gradient of solvent B between 5% and 65% at a flow rate of 3 ml/min. Analytical RP-HPLC was performed on a Kromasil C_18_ column (250 mm × 4.6 mm, 5 μm, 100 Å; dichrom GmbH, Marl, Germany) using a linear gradient of solvent B between 5% and 65% at a flow rate of 1 ml/min. The eluting peptides were monitored by UV absorbance at 214, 254, and 280 nm wavelengths. Cyclotides in *V. tricolor* were identified by molecular weight and retention time by comparison to CyBase (www.cybase.org.au; Wang et al., 2007) and data published in Hellinger et al. (Hellinger et al., 2015).

### Cell Culture

The reporter cell line TZM-bl (also called JC53-bl; NIH ARRRP, ARP-8129) and HEK293T (human embryo kidney; DSMZ, ACC-635) cells were cultivated in DMEM which was supplemented with 10% heat-inactivated fetal calf serum (FCS), 100 units/ml penicillin, 100 μg/ml streptomycin and 2 mM l-glutamine. Cells were grown at 37°C in a 5% CO_2_ humidified incubator, were regularly tested for mycoplasma contamination and remained negative.

### HIV-1 Production

Virus stock of the CCR5-tropic HIV-1 NL4-3 92TH014-12 (Papkalla et al., 2002) was generated by transient transfection of 293T cells with proviral constructs as described (Münch et al., 2007). Transfection mixture was replaced by 2 ml DMEM supplemented with 2 mM l-glutamine, 100 units/ml penicillin, and 100 mg/ml streptomycin and 2.5% heat-inactivated FCS after overnight incubation. 2 days later, virus was collected by centrifuging the cell supernatant for 3 min at 330 x g to remove cell debris. Virus stocks were stored at −80°C. The amount of virus to be used in infectivity assays was determined by titration.

### HIV-1 Infection Assay

HIV-1 infection was quantified using TZM-bl reporter cells stably transfected with an LTR-lacZ cassette (Wei et al., 2002). Upon infection with HIV-1 the viral protein Tat is expressed, which activates the LTR promotor and results in β-galactosidase expression. For infection assays, 10,000 TZM-bl cells were seeded the day before into 96-well plates. The next day, the medium was replaced with serumfree X-vivo 15 medium (Lonza, BE02-060F). For cell treatment, cells were treated with the titrated compounds and afterward infected. For virus treatment, virus and titrated compounds were mixed and preincubated and subsequently added onto the cells. 2 days later, infection rates were determined by detecting the β-galactosidase activity in cellular lysates using the gal-screen β-galactosidase reporter gene assay system for mammalian cells (Thermo Fisher Scientific) and the Orion II microplate luminometer (Titertek Berthold). Measured values represent reporter gene activity (RLU/s) and were corrected for the background signal derived from uninfected cells. Untreated controls were set to 100% infection.

### Cell Viability Assay

To assess toxicity of tested compounds, assays were performed in parallel to infection assays using medium instead of virus. 2 days later, metabolic activity was quantified via an MTT-based assay. To this end, medium was removed and 100 µl of 1:10 diluted MTT solution (Sigma Aldrich) was added. After 2.5 h at 37°C, supernatant was removed and 100 µl of DMSO-ethanol (1:1) solution was added to dissolve formazan crystals. Then, absorption was measured at 570 nm and baseline corrected for 650 nm using the Vmax Kinetic ELISA microplate reader (Molecular Devices, LLC). Untreated controls were set to 100%.

### Bright-Field Microscopy

Cells were treated like for cell viability assessment. 2 days later, images of the cells were taking using a Cytation™ 3 cell imaging system and processed with Gen5 (BioTek).

### Nonlinear Regression

The determination of the inhibitory concentration 50 (IC_50_) and cell toxic concentration 50 (CC_50_) were calculated by nonlinear regression ([Inhibitor] vs. normalized response -- Variable slope) in GraphPad Prism Version 9.2.0 for Windows, GraphPad Software, San Diego, California United States, www.graphpad.com. These values were used to calculate the selectivity index (SI) by dividing CC_50_ by IC_50_.

## Results

### Screening of Cysteine-Rich Plant Extracts for Anti-HIV Activity

**FIGURE 2:**
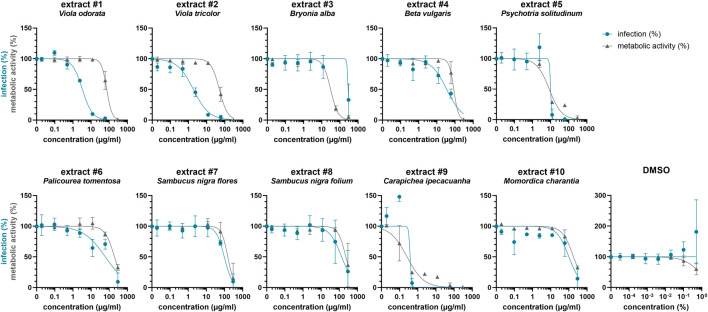
Effect of plant extracts on HIV-1 infection of TZM-bl cells. Ten peptide-enriched plant extracts were obtained using solvent and solid phase extraction, and their molecular content was analyzed by MALDI-TOF MS (Figure S1). Extracts were dissolved in 5% DMSO. Serial dilutions of extracts #1 to #10 and DMSO control were added to TZM-bl cells, which were then infected with HIV-1 (infection, blue) or medium was added (metabolic activity, grey). After 2 days, infection and metabolic activity was quantified via β-galactosidase or MTT-based assay, respectively. Untreated controls were set to 100%. Shown are means ± SD of one (#10, metabolic activity), two (#10, infection) or three (all others) independent experiments performed in triplicates.

Driven by previous findings that cysteine-rich peptides exhibit anti-HIV properties (Bokesch et al., 2001; Ireland et al., 2008; Wang et al., 2008), we established a new library of plant extracts comprising cysteine-rich peptides, as previously described (Muratspahić et al., 2021). This library largely consisted of plants with ethnopharmacological relevance that have previously been identified as a rich source of cyclotides such as *Viola odorata*, *Viola tricolor*, *Psychotria solitudinum*, *Palicourea tomentosa* and *Carapichea ipecacuanha* (Koehbach et al., 2013; Fahradpour et al., 2017; de Veer et al., 2019). Furthermore, the plant extract library included species that are rich in other cyclotide-like or knottin peptides such as *Bryonia alba*, *Beta vulgaris*, *Sambucus nigra* as well as *Momordica charantia* (He et al., 2013; Karpyuk et al., 2015; Álvarez et al., 2018; Retzl et al., 2020). Using solvent and solid phase extraction yielded the plant extracts enriched in cysteine-rich peptides. As quality control of the molecular content of peptide-enriched extracts MALDI-TOF MS analysis was used, which confirmed the presence of peptide mass signals in the range of 2,500–4,000 Da (Supplementary Figure S1). Subsequently, these extracts were tested for antiviral activity against HIV-1 by adding serial dilutions of the extracts to TZM-bl cells, which were then infected with HIV-1. All extracts reduced HIV-1 infection rates dose-dependently, however they also reduced metabolic activity (Figure 2). Hence, the 50% inhibitory concentration (IC_50_) as well as the 50% cytotoxic concentration (CC_50_) was calculated and used to determine the selectivity index (SI) which is the ratio of the toxic concentration of a sample against its effective bioactive concentration (Table 1). This revealed IC_50_ values between 0.4 ± 0.1 μg/ml and 278.7 ± 31.8 μg/ml, but SIs for extracts #3 to #10 were low. In contrary, extracts #1 (*Viola odorata*) and #2 (*Viola tricolor*) had low IC_50_ values of 3.3 ± 0.1 μg/ml and 1.8 ± 0.7 μg/ml, which yielded good SIs of 22.0 ± 3.9 and 31.8 ± 17.3, respectively.

**TABLE 1:** Summary of antiviral activity of plant extracts.

Extract #	Plant	CC50 (µg/ml)*	IC50 (µg/ml)*	SI#
1	Viola odorata	72.7 ± 12.6	3.3 ± 0.1	22.0 ± 3.9
2	Viola tricolor	51.0 ± 12.7	1.8 ± 0.7	31.8 ± 17.3
3	Bryonia alba	30.8 ± 1.0	278.7 ± 31.8	0.1 ± 0.0
4	Beta vulgaris	89.6 ± 22.7	32.5 ± 9.7	2.9 ± 0.8
5	Psychotria solitudinum	8.2 ± 0.7	9.9 ± 0.8	0.8 ± 0.1
6	Palicourea tomentosa	196.4 ± 25.7	70.9 ± 51.6	3.9 ± 2.4
7	Sambucus nigra flores	161.1 ± 114.9	90.4 ± 23.9	1.7 ± 0.8
8	Sambucus nigra folium	214.8 ± 73.8	137.1 ± 141.3	2.5 ± 1.4
9	Carapichea ipecacuanha	0.2 ± 0.1	0.4 ± 0.1	0.6 ± 0.1
10	Momordica charantia	181.4	112.1 ± 35.2	1.3

*CC50 and IC50 were determined by nonlinear regression from concentration-response curves (Figure 2). Values represent means ± SD of three independent experiments in triplicates with the exception of extract #10 (only one (metabolic activity) or two (infection) independent experiments).

#SI, was calculated by dividing CC50 by IC50.

### Cyclotide-Enriched Fractions of *V. tricolor* Exhibit Anti-HIV Activity

**FIGURE 3:**
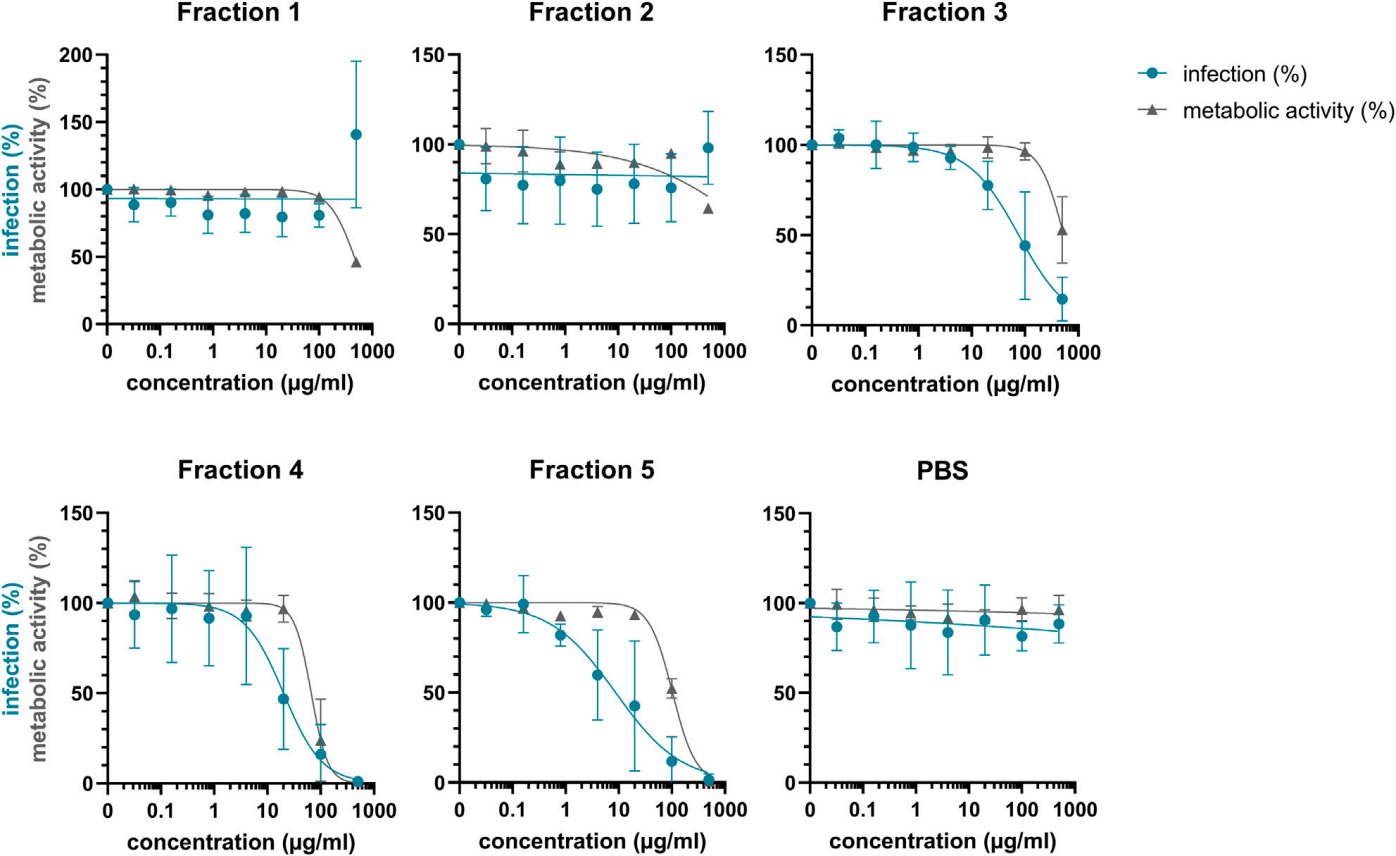
Anti-HIV-1 activity of *Viola tricolor* peptide-enriched fractions. Extract #2 (*V. tricolor*) was fractionated and fractions dissolved in PBS. Then, fractions 1-5 and PBS control were titrated and added to TZM-bl cells. Cells were infected with HIV-1 (infection, blue) or medium was added (metabolic activity, grey). 2 days later, infection and metabolic activity was quantified via β-galactosidase or MTT-based assay, respectively. Data are normalized to untreated controls and represented as means ± SD of three independent experiments in triplicates.

Considering that extract #2 (*V. tricolor*) exhibited the most pronounced anti-HIV effect and had the best SI, we next conducted a multistep bioactivity-guided fractionation of this extract to identify the peptide(s) with anti-HIV properties. Accordingly, we performed preparative RP-HPLC thereby generating five distinct cyclotide-enriched fractions, referred to as fractions 1–5 (Supplementary Figure S2). These fractions were characterized by analytical RP-HPLC and MALDI-TOF MS to confirm the presence of cyclotides (Supplementary Table S1) and subsequently tested for anti-HIV-1 activity. Fraction 1 and 2 had no effect, whereas fraction 3-5 inhibited HIV-1 in concentration-dependent manner with IC_50_ values of 111.1 ± 114.2 μg/ml, 21.0 ± 18.3 μg/ml and 20.5 ± 28.2 μg/ml, respectively (Figure 3; Table 2). Since they also reduced the metabolic activity, the SI was calculated again, showing highest selectivity for fraction 5 with a SI of 18.6 ± 16.4, but also good selectivity for fraction 3 and 4 with SIs of 8.3 ± 6.7 and 11.7 ± 15.5, respectively (Table 2).

**TABLE 2:** Summary of the antiviral activity of *Viola tricolor* peptide-enriched fractions.

Fraction #	CC50 (µg/ml)*	IC50 (µg/ml)*	SI#
1	464.3 ± 31.9	>500	<0.9 ± 0.1
2	>500	>500	nd
3	485.8 ± 123.9	111.1 ± 114.2	8.3 ± 6.7
4	73.8 ± 32.5	21.0 ± 18.3	11.7 ± 15.5
5	103.2 ± 9.5	20.5 ± 28.2	18.6 ± 16.4

*CC50 and IC50 were determined by nonlinear regression from concentration-response curves (Figure 3). Values represent means ± SD of three independent experiments in triplicates. Since 500 μg/ml was the highest concentrations used, no higher half-maximal concentrations could be calculated.

#SI, was calculated by dividing CC50 by IC50. nd: not determined.

**FIGURE 4:**
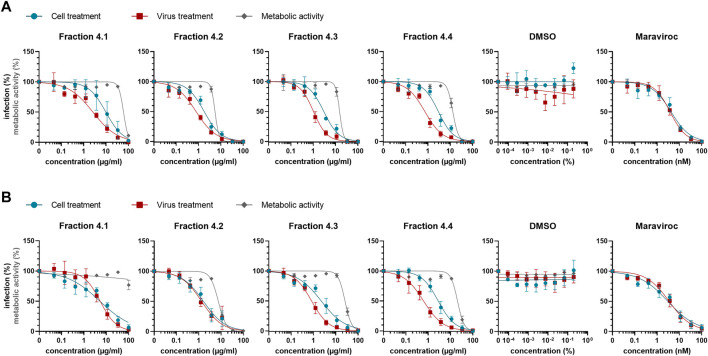
Anti-HIV-1 activity of *Viola tricolor* peptide subfractions. Fraction 4 of extract #2 (*V. tricolor*) was further fractionated and fractions dissolved in 5% DMSO. **(A)** Fractions 4.1–4.4 and controls (solvent control DMSO and inhibitor maraviroc) were titrated and added onto seeded TZM-bl cells in serumfree X-vivo medium, incubated for 30 min and subsequently infected with HIV-1 (cell treatment, blue). Alternatively, subfractions were first incubated with virus for 30 min. Then, these mixtures were used to infect the cells (virus treatment, red). 2 dpi, infection was quantified by β-galactosidase assay. In parallel, cell viability was quantified using MTT-based assay (metabolic activity, grey). **(B)** Experiments were performed like in A described. Additionally, 3 hpi medium was replaced by fresh compound-free medium. Data in A and B are normalized to untreated controls and represented as means ± SD of three independent experiments in triplicates.

Since the cyclotide-enriched fractions 3-5 showed antiviral activity, all three will most likely contain antiviral cytlotides and could be used for future studies. For this study, we opted for fraction 4 and further generated four sub-fractions by semipreparative RP-HPLC fractionation, namely fractions 4.1–4.4 (Supplementary Figure S3). These fractions contained several cyclotides, which were identified and characterized by MALDI-TOF MS and RP-HPLC (Table 3, Supplementary Table S2) including vigno 5, acyclic vitri E, acyclic cO22, cO28 (fraction 4.1), vitri peptide 2, vigno 9, cO2 (fraction 4.2), kalata S, varv C/D, acyclic vigno 5 (fraction 4.3) and kalata B1, varv E, vigno 3, vigno 4, chacur 1, and cO22 (fraction 4.4). The four fractions were then analyzed using the same experimental conditions as described above (“cell treatment”). In parallel, the virus was pre-incubated with the titrated subfractions and then used to infect the cells (“virus treatment”) reaching the same final concentrations as under cell treatment conditions. In line with the observed activity of mother fraction 4, semipure *V. tricolor* cyclotide fractions 4.1–4.4 displayed anti-HIV activity and also reduced metabolic activity of the treated cells under both experimental conditions (Figure 4A, Supplementary Figure S4). The IC_50_ values were in the range between 0.7 ± 0.1 μg/ml and 11.2 ± 9.0 μg/ml. The highest activity was observed for virus pretreatment with fraction 4.4 with an IC_50_ of 0.7 ± 0.1 μg/ml (Table 4). The best selectivity had fraction 4.1 with a SI of 8.1 ± 5.4. All four subfractions where more active in virus than in cell treatment, suggesting that the inhibitory effect is directed against the viral particle. In contrary, the control inhibitor maraviroc, an entry inhibitor blocking the HIV receptor CCR5, had similar antiviral activities under both experimental conditions (Figure 4A; Table 4).

**TABLE 3:** Overview about tested *Viola tricolor* cyclotide subfractions.

Fraction #	Cyclotides	Mass monoiso. (m/z)	Retention time (min)	Purity (%)
4.1	vigno 5	2,858.8	46.67	>70
	acyclic vitri E, acyclic cO22, cO28	2,922.8		
4.2	vitri peptide 2, vigno 9, cO2	3,138.9	44.20	>80
4.3	kalata S, varv C/D, acyclic vigno 5	2,876.7	47.65	>90
4.4	kalata B1, varv E, vigno 3	2,890.6	48.77	∼50
	vigno 4, chacur 1, cO22	2,904.6		

Cyclotides in each subfraction were identified by molecular weight obtained by MALDI-TOF MS, as compared to CyBase entries for Viola tricolor (Wang et al., 2007).

**TABLE 4:** Overview about anti-HIV-1 activity of *Viola tricolor* peptide subfractions.

Fr. #	Without washing				With washing			
	IC50 (µg/ml or nM)		CC50 (µg/ml)	SI (CT)	IC50 (µg/ml or nM)		CC50 (µg/ml)	SI (CT)
	VT	CT			VT	CT		
4.1	2.6 ± 0.4	11.2 ± 9.0	60.4 ± 1.9	8.1 ± 5.4	5.2 ± 2.0	5.3 ± 2.3	>100	>21.7 ± 10.8
4.2	0.8 ± 0.2	1.8 ± 0.3	5.5 ± 0.3	3.1 ± 0.3	1.4 ± 0.4	1.7 ± 0.8	7.5 ± 0.4	4.9 ± 2.0
4.3	1.1 ± 0.3	2.9 ± 0.7	15.4 ± 1.3	5.5 ± 1.2	0.8 ± 0.0	2.4 ± 1.2	25.9 ± 2.1	12.5 ± 5.3
4.4	0.7 ± 0.1	3.0 ± 0.5	12.9 ± 1.0	4.4 ± 0.4	0.6 ± 0.2	2.9 ± 0.9	20.6 ± 0.9	7.5 ± 1.8
mara.	3.8 ± 1.0	4.3 ± 1.8	-	-	3.7 ± 1.4	3.3 ± 1.2	-	-

CC50 and IC50 were determined by nonlinear regression from concentration-response curves (Figure 4) and are given in µg/ml units for fractions and nM units for maraviroc. Values represent means ± SD of three independent experiments in triplicates. SI was calculated by dividing CC50 by IC50. Since 100 μg/ml was the highest concentrations used, no higher half-maximal concentrations could be calculated. CT, cell treatment; VT, virus treatment; mara, maraviroc.

As the antiviral activity is directed against the virus particles, we were wondering whether a reduced incubation time of the fractions with the cells may result in similar antiviral effects. To test this, we repeated the experiments using both, cell and virus-treatment conditions, but removed the viral inoculum and the compounds 3 hours post infection. Cells were then further cultivated in pure medium, and cytotoxicity and HIV-1 infection rates were determined 2 days later as before. As shown in Figure 4B, the antiviral activities were in the same range (IC_50_s between 0.6 ± 0.2 μg/ml and 5.3 ± 2.3 μg/ml; Table 4) as compared to Figure 4A, further indicating that the inhibitory activity is rapidly directed against the virus. In contrary, cell toxicity was reduced (Figure 4B, Supplementary Figure S5).

## Discussion

Plant defense peptides such as cyclotides are thought to be part of the innate plant host defense. The unique disulfide-knotted and head-to-tail cyclized topology may be one reason for the previously observed bioactivity of cyclotides (Daly et al., 2004; Ireland et al., 2008; Wang et al., 2008; Gerlach et al., 2019). To identify novel antiviral peptides, we established a library of plant extracts largely consisting of cyclotide-rich species with ethnopharmacological relevance and screened them against HIV-1, a representative and important member of the retrovirus family. Following the initial screen, the most active extract of *Viola tricolor* was selected to detailed analysis. Through two HPLC purification steps, four *Viola tricolor*-derived peptide subfractions were obtained which inhibited HIV-1 concentration-dependently with IC_50_ values between 0.6 and 9.8 μg/ml. The antiviral fractions contained several cyclotides such as vigno 2/5, vitri 2, vigno 8/9, kalata S, vigno 3/4, kalata B1, and cycloviolacin O12/O22, belonging to the Möbius or the bracelet subfamily (Table 3, Supplementary Table S2). Cyclotides have been found in every member of the Violaceae family examined so far and they occur in all tissues of these plants, including roots, stems, leaves and flowers (Burman et al., 2015). These cyclotides have previously been identified in different *Viola* species (Ireland et al., 2006; Hashempour et al., 2013; Hellinger et al., 2015; Esmaeili et al., 2016), which is not surprising since more than 200 cyclotides are known within the Violaceae family (de Veer et al., 2019). However, only for some anti-HIV activity was shown to date (Gustafson et al., 1994; Daly et al., 2004, 2006; Wang et al., 2008).

All subfractions tested were more active when the virus was pre-incubated, suggesting that antiviral activity is not mediated indirectly *via* a cell factor, but by the direct action of cyclotides on viruses. When the compounds were washed out 3 hours after infection, the antiviral activities were in the same range as when treated for 2 days, which is further evidence that the antiviral activity is mediated by the actual effect on the virions. This is in line with previously published data showing that cyclotides are able to permeabilize model membranes (Henriques and Craik, 2012; Henriques et al., 2012). It has been published that cyclotides target membranes through specific interactions with phospholipids containing phosphatidylethanolamine (PE) headgroups and then insert through nonspecific hydrophobic peptide-lipid interactions (Henriques et al., 2011, 2012; Henriques and Craik, 2012; Cranfield et al., 2017). Subsequently, this promotes outward movement of PE phospholipids, exposing more PE in the outer leaflet. This self-promotes the binding of more cyclotides until a threshold concentration is achieved leading to self-aggregation, pore formation and eventual membrane disruption (Henriques and Craik, 2012). Whether the cyclotides identified in this work may exert similar effects needs to be further examined.

The analyzed cyclotide fractions also reduced metabolic activity, which is in line with previous studies (Ireland et al., 2008; Wang et al., 2008). This effect on the cells can probably be explained by the mechanism of action of the cyclotides acting on lipid membranes (Henriques and Craik, 2012). However, the activity of cyclotides is dependent on their affinity for lipid bilayers and the lipid composition. Although the total lipid in mammalian cells comprises ∼20% PE phospholipids (Patel and Witt, 2017), model membrane studies showed that kalata B1 targets HIV particles with high preference, since raft-like HIV membranes contain PE phospholipids and cholesterol/sphingomyelin domains, which enhances the interaction (Brügger et al., 2006; Henriques et al., 2011; Mücksch et al., 2019). The preference for HIV particles over eukaryotic cells is also supported by the calculated selectivity index (SI) of up to 8.1 ± 5.4 herein. Such therapeutic windows have also been observed by others (Ireland et al., 2008; Wang et al., 2008), e.g. Wang et al. determined a CC_50_ of 5.7 µM and an IC_50_ of 0.66 µM resulting in an SI of 8.6. The ideal compound should have a relatively high toxic but a very low active concentration. The higher the SI ratio, the theoretically more effective and safer a drug would be during *in vivo* treatment (Cushnie et al., 2020). Besides, the hemolytic activity of several cyclotides isolated from *V. tricolor* has been determined previously with 50% hemolytic concentrations (HD_50_ values) ranging from 4 to 226 µM suggesting a similar or even higher SI for red blood cell lysis (Tang et al., 2010).

In the future, it would be interesting to investigate if cyclotides have preferences for specific vesicles sizes. The so-called curvature-sensing peptides such as the AH-peptide preferentially target smaller vesicles and are antiviral (Jackman et al., 2018; Park et al., 2019). Membrane bending is enlarged in smaller vesicles, leads to lipid packaging defects between neighboring lipid molecules and subsequently to insertion of the amphipathic α-helical peptides (Kawano et al., 2019; Park et al., 2019). It cannot be expected that cyclotides have the same mechanism since they have a different tertiary structure, however, stronger membrane bending in smaller vesicles leading to packaging defects may also facilitate hydrophobic peptide-lipid interactions, pore formation and membrane disruption. This could lead to the preferential destruction of viral over cellular membranes improving the therapeutic window.

It is noteworthy that the antiviral fractions contained several cyclotides, which raises the question of synergistic effects. A well-known advantage of plant substances is their complex composition, consisting of many compounds with multiple activities that together give a greater overall activity (Schmidt et al., 2008). Also, typical AMPs can have synergistic activity (Lazzaro et al., 2020). Therefore, mixtures of cyclotides could also have additive antiviral effects that reduce the tendency to develop resistance. Moreover, Gerlach et al. showed that the cyclotide cycloviolacin O2 increases the activity of the HIV protease inhibitors saquinavir and nelfinavir by pore-formation in HIV-infected cells and viral membranes at non-hemolytic concentrations (Gerlach et al., 2013, 2019). It is also suggested to use AMPs in combination therapies to have higher activity but also to reduce resistance development (Dijksteel et al., 2021). Hence, cyclotides may augment antiretroviral therapy efficacy and be useful for combination therapies.

Since cyclotides act on lipid membranes it is reasonable to assume that they could be broadly effective antiviral agents. Indeed, the antiviral activity of a cyclotide against influenza A virus has been reported (Liu et al., 2014) and it would be interesting to investigate the antiviral activity of the extracts or purified cyclotides against other viral pathogens such as SARS-CoV-2. In addition, cyclotides have been successfully used for molecular grafting, i.e. inserting small bioactive epitopes into the stable cyclotide scaffold (Wang et al., 2014). Due to the plasticity of cyclotides, this usually preserves their structural integrity and activity (Clark et al., 2006) and can enable the development of dual-function antiviral agents by conjugating the cyclotides with specific virus entry inhibitors.

In sum, cyclotides and extracts described herein may offer a promising starting point for innovative therapeutic antiviral agents: the peptides have been structurally optimized by evolution to serve particular biological functions, and can be optimized for activity and stability for therapeutic applications (Atanasov et al., 2021). Moreover, cyclotides have an exceptional resistance to thermal, chemical and enzymatic degradation (Colgrave and Craik, 2004) which might increase bioavailability, and their mode of action has limited propensity for resistance development (Mahlapuu et al., 2016; Browne et al., 2020; Huan et al., 2020). Above all, *V. tricolor* is a medicinal plant, listed in the European Pharmacopoeia (Hellinger et al., 2015; EMA/HMPC/131734/2009), and its herein described properties may be explored in the future for applications of herbal preparations with antiviral activity.

## Data Availability

The raw data supporting the conclusion of this article will be made available by the authors, without undue reservation.
