# Supplementary material for: In vitro Inhibition of HIV-1 by Cyclotide-Enriched Extracts of Viola tricolor
Source: Front Pharmacol. 2022 May 27;13:888961. doi: 10.3389/fphar.2022.888961 (PMC9196940; doi:10.3389/fphar.2022.888961)
Supplement: Supplementary file 1 [file DataSheet1.docx]

Supplementary Material

# Supplementary Figures

**
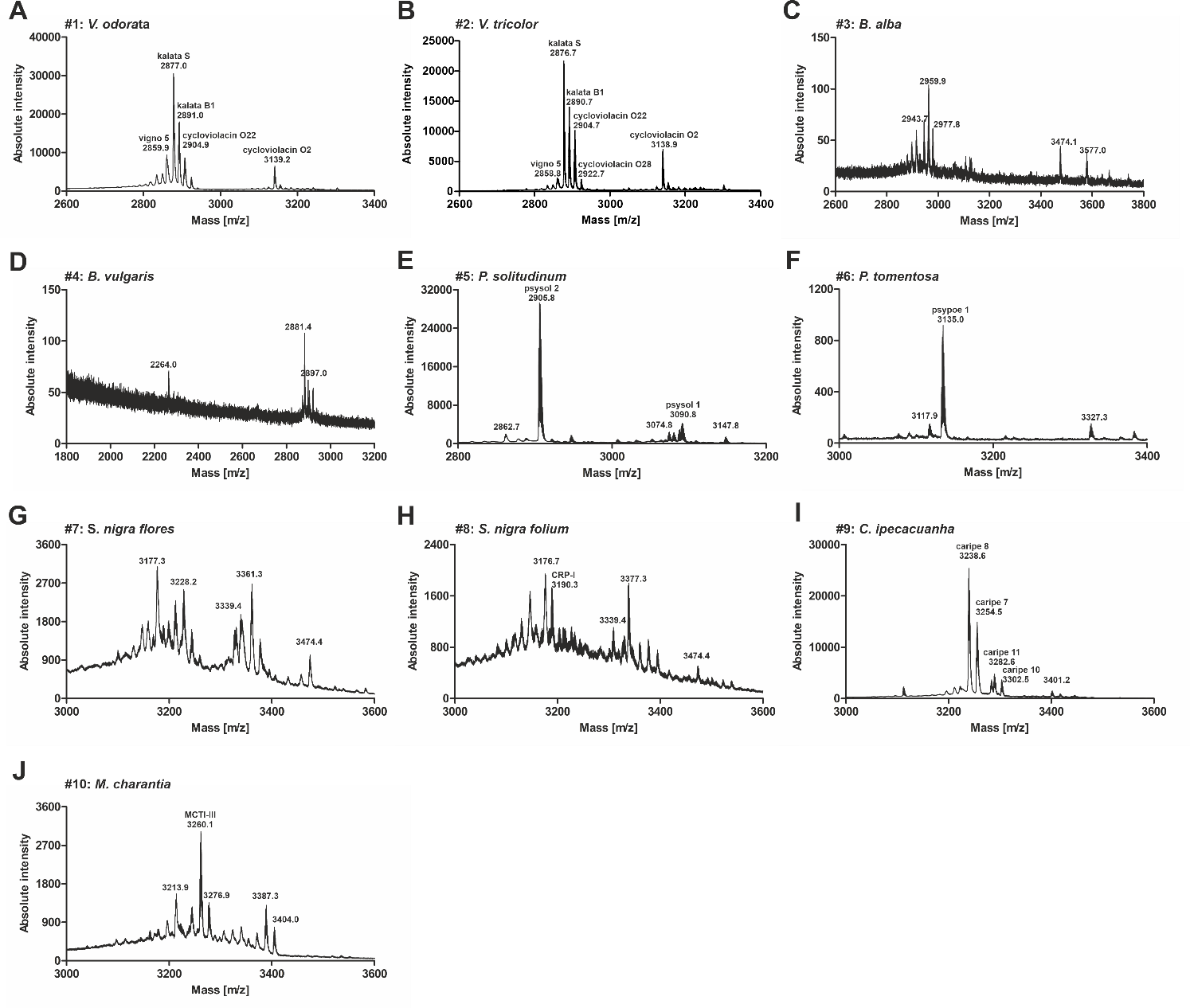
**

**Supplementary Figure S1: Analytical profiles of cysteine-rich plant extracts.** (A)-(J) MALDI-TOF mass spectra of cysteine-rich extracts of *V. odorata* (A), *V. tricolor* (B) *B. alba* (C), *B. vulgaris* (D), *P. solitudinum* (E), *P. tomentosa* (F), *S. nigra flores* (G), *S. nigra folium* (H), *C. ipecacuanha* (I) and *M. charantia* (J). The major mass signals are denoted as monoisotopic masses [M+H]^+^. Matching monoisotopic masses (± 1 m/z) were labelled with the corresponding cyclotide name assigned in CyBase (Wang et al., 2007).

**
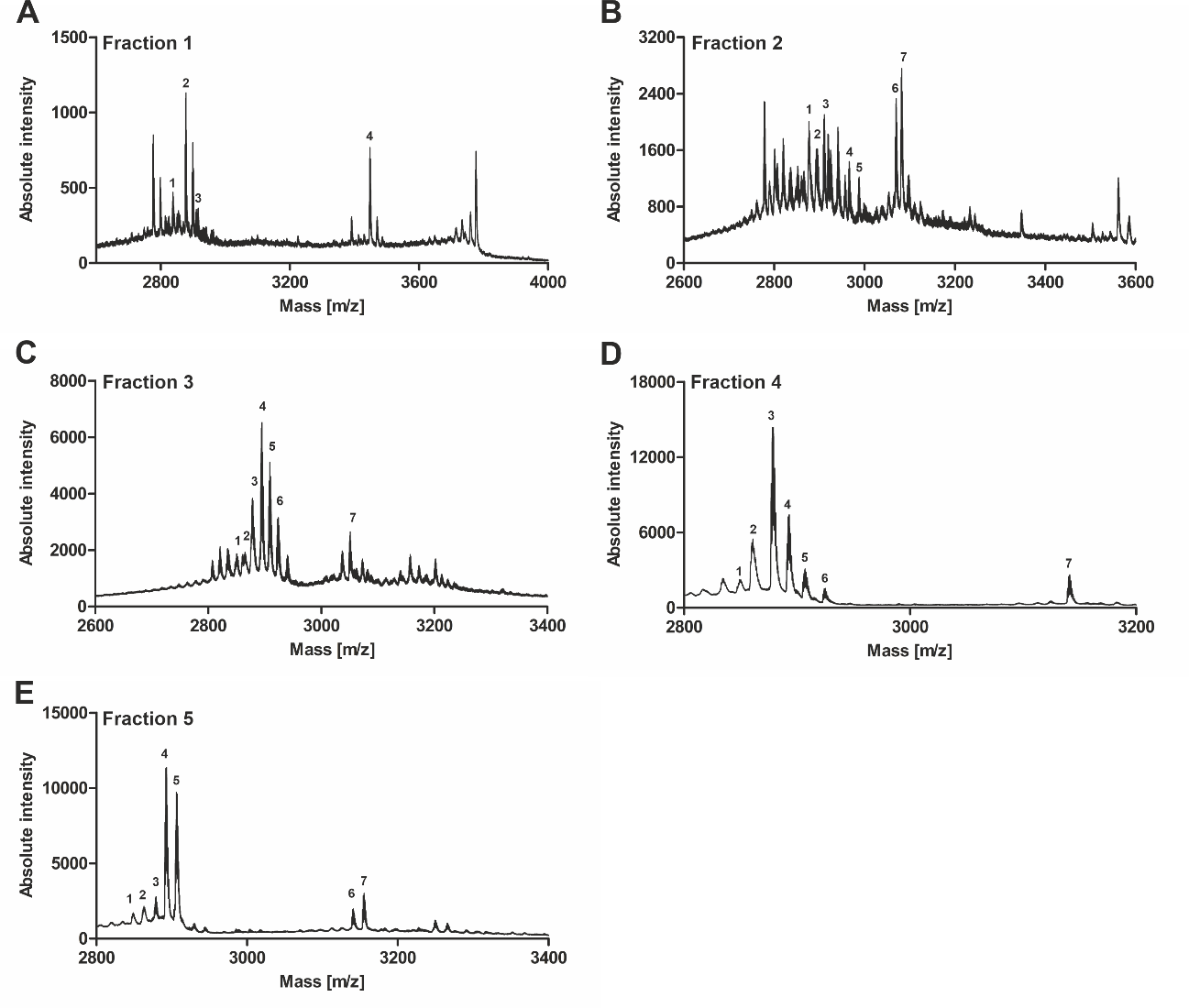
**

**Supplementary Figure S2: HPLC fractionation of *V. tricolor* peptide-enriched extract.** (A)-(E) MALDI-TOF mass spectra of fractions containing cysteine-rich peptides of *V. tricolor* extract. Denoted major mass signals were compared to known cyclotide masses of *V. tricolor* published in the CyBase database (Wang et al., 2007) and by Hellinger et al. (Hellinger et al., 2015). The respective cyclotides are listed in Table S1.


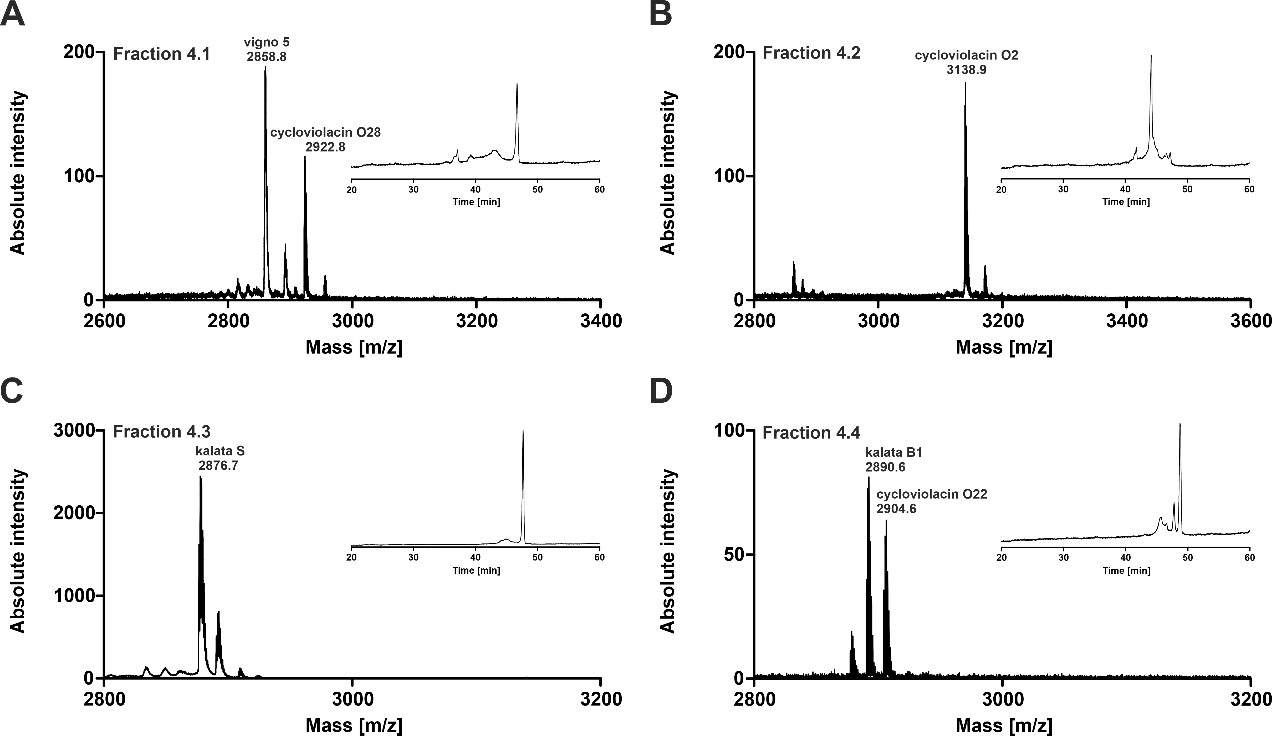


**Supplementary Figure S3: Analytical profile of cyclotide-enriched subfractions.** (A)-(D) MALDI-TOF mass spectra and RP-HPLC chromatograms (small insets) of cyclotide-enriched fractions 4.1-4.4. Molecular weights are denoted as monoisotopic masses [M+H]^+^ and were assigned to known cyclotides of *V. tricolor* published in the CyBase database (Wang et al., 2007).


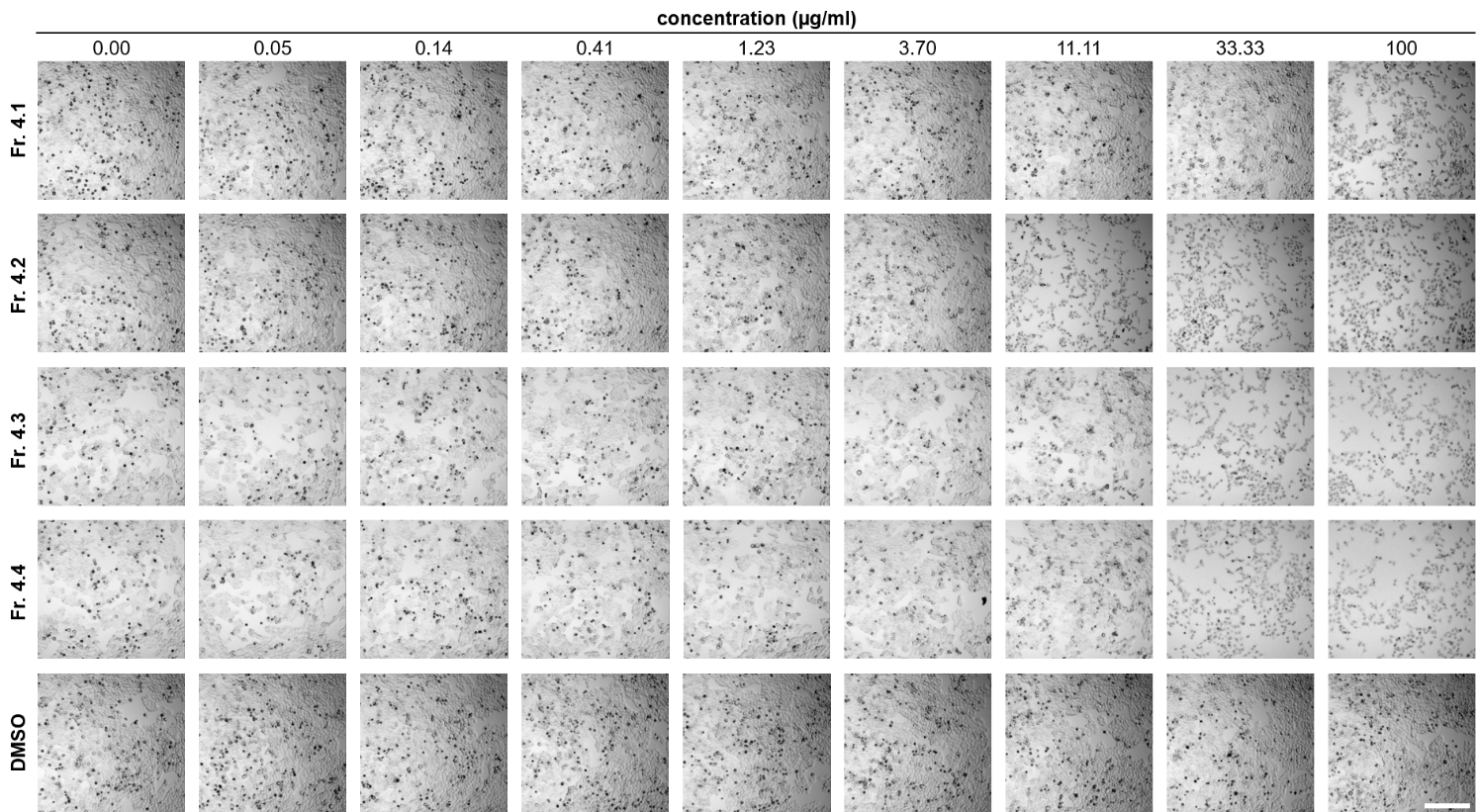


**Supplementary Figure S4: Bright-field microscopy of TZMbl cells treated with *V. tricolor* peptide subfractions for 2 days**. TZMbl cells were treated with serial dilutions of fractions 4.1-4.4. After 2 days, cells were monitored by bright field microscopy using a Cytation™ 3 cell imaging system. Scale bar represents 300 µm.


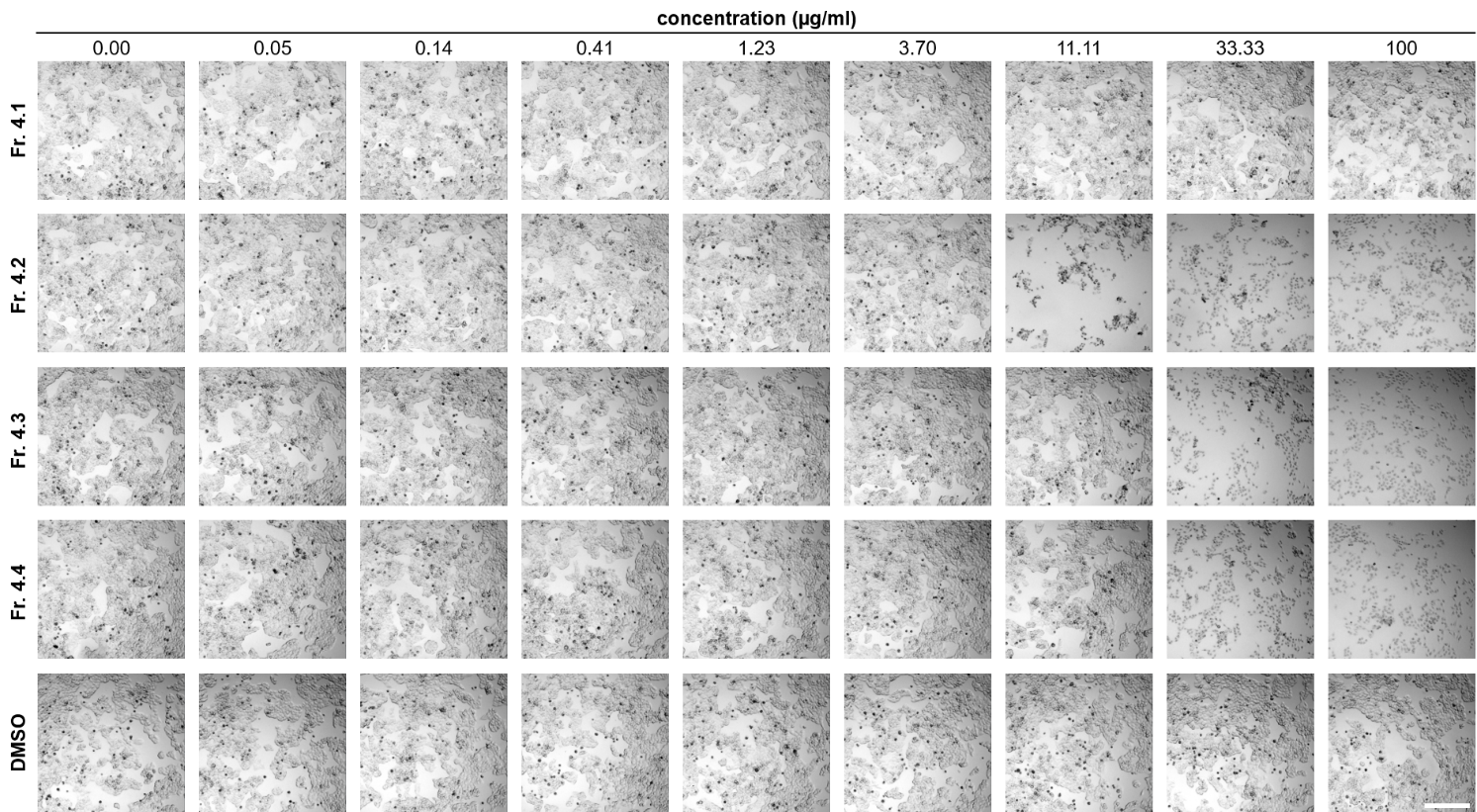


**Supplementary Figure S5: Bright-field microscopy of TZMbl cells treated with *V. tricolor* peptide subfractions for 3 hours**. TZMbl cells were treated with serial dilutions of fractions 4.1-4.4 and 3 hours later, medium was replaced with fresh compound-free medium. After 2 days, cells were monitored by bright field microscopy using a Cytation™ 3 cell imaging system. Scale bar represents 300 µm.

# Supplementary Tables

**Supplementary Table S1: Identified cyclotides in *V. tricolor* fractions**

| Fraction No. | Peak No. | Mass [m/z]* | Cyclotide |
| --- | --- | --- | --- |
| 1 | 1 | 2836.1 | vitri 41 |
|  | 2 | 2876.1 | acyclic vigno 5, kalata S, varv C, varv D |
|  | 3 | 2908.1 | acyclic kalata B1, varv peptide E, acyclic vigno 3 |
|  | 4 | 3446.5 | vitri 21 |
| 2 | 1 | 2875.2 | kalata S, varv C, varv D, acyclic vigno 5 |
|  | 2 | 2891.2 | [kalata B1](http://www.cybase.org.au/index.php?page=card&table=protein&id=1), cO12, [vigno 3](http://www.cybase.org.au/index.php?page=card&table=protein&id=784), [vitri 94a](http://www.cybase.org.au/index.php?page=card&table=protein&id=1766) |
|  | 3 | 2908.6 | acyclic kalata B1, varv peptide E, acyclic vigno 3 |
|  | 4 | 2964.2 | vitri C |
|  | 5 | 2986.2 | vitri 23 |
|  | 6 | 3068.3 | varv peptide B |
|  | 7 | 3080.3 | kalata B3, vibiE, mram11, cO29 |
| 3 | 1 | 2846.1 | viba 32 |
|  | 2 | 2859.2 | [viba 15](http://www.cybase.org.au/index.php?page=card&table=protein&id=386), vigno 5 |
|  | 3 | 2875.2 | kalata S, varv C, varv D |
|  | 4 | 2893.1 | acyclic kalata S |
|  | 5 | 2907.1 | acyclic kalata B1, acyclic vigno 3, varv peptide E |
|  | 6 | 2921.1 | vitri E, acyclic cO22, cO28 |
|  | 7 | 3049.3 | vitri peptide 39, vaby E |
| 4 | 1 | 2847.2 | viba 32 |
|  | 2 | 2859.1 | [viba 15](http://www.cybase.org.au/index.php?page=card&table=protein&id=386), vigno 5 |
|  | 3 | 2877.2 | kalata S, varv C, varv D, acyclic vigno 5, acyclic viba 30 |
|  | 4 | 2891.2 | [kalata B1](http://www.cybase.org.au/index.php?page=card&table=protein&id=1), cO12, [vigno 3](http://www.cybase.org.au/index.php?page=card&table=protein&id=784), [vitri 94a](http://www.cybase.org.au/index.php?page=card&table=protein&id=1766) |
|  | 5 | 2905.1 | cO22, chacur 1, vigno 4 |
|  | 6 | 2923.1 | acyclic cO22, cO28, vitri E |
|  | 7 | 3139.4 | cO2, vigno 9, vitri peptide 2 |
| 5 | 1 | 2847.2 | viba 32 |
|  | 2 | 2861.2 | [viba 15](http://www.cybase.org.au/index.php?page=card&table=protein&id=386), viba 30 |
|  | 3 | 2877.1 | acyclic viba 30, acyclic vigno 5, kalata S, varv C, varv D |
|  | 4 | 2891.1 | cO12, [kalata B1](http://www.cybase.org.au/index.php?page=card&table=protein&id=1), [vigno 3](http://www.cybase.org.au/index.php?page=card&table=protein&id=784), [vitri 94a](http://www.cybase.org.au/index.php?page=card&table=protein&id=1766) |
|  | 5 | 2905.1 | cO22, chacur 1, vigno 4 |
|  | 6 | 3139.3 | cO2, vigno 9, vitri peptide 2 |
|  | 7 | 3153.4 | cO20, vitri A/cO3 |
| *Peak masses derived from MALDI-TOF were compared to known cyclotides masses of *V. tricolor* published in the CyBase database (Wang et al., 2007) and by Hellinger et al. (Hellinger et al., 2015). | | | |

**Supplementary Table** **S2: Identified cyclotides in *V. tricolor* subfractions**

| Fr. # | Cyclotide | Sequence | Net charge  (pH 7) | Ratio of hydrophilic residues (%) |
| --- | --- | --- | --- | --- |
| 4.1 | vigno 5 | cyclo-GLPLCGETCVGGTCNTPGCSCGWPVCVRN | -0.27 | 17 |
|  | acyclic vitri E | GLPVCGETCVGGTCNTPGCSCSWPVCFRN | -0.27 | 21 |
|  | acyclic cO22 | GLPICGETCVGGTCNTPGCTCSWPVCTRN | -0.27 | 17 |
|  | cO28 | cyclo-GLPVCGETCVGGTCNTPGCSCSWPVCFRD | -1.27 | 21 |
| 4.2 | vitri peptide 2 | cyclo-GSIPCGESCVWIPCISGIAGCSCSNKVCYLN | -0.27 | 29 |
|  | vigno 9 | cyclo-GIPCGESCVWIPCISSALGCSCKSKVCYRN | 1.73 | 33 |
|  | cO2 | cyclo-GIPCGESCVWIPCISSAIGCSCKSKVCYRN | 1.73 | 33 |
| 4.3 | kalata S | cyclo-GLPVCGETCVGGTCNTPGCSCSWPVCTRN | -0.27 | 21 |
|  | varv C | cyclo-GVPICGETCVGGTCNTPGCSCSWPVCTRN | -0.27 | 21 |
|  | varv D | cyclo-GLPICGETCVGGSCNTPGCSCSWPVCTRN | -0.27 | 24 |
|  | acyclic vigno 5 | GLPLCGETCVGGTCNTPGCSCGWPVCVRN | -0.27 | 17 |
| 4.4 | kalata B1 | cyclo-GLPVCGETCVGGTCNTPGCTCSWPVCTRN | -0.27 | 17 |
|  | varv E | cyclo-GLPICGETCVGGTCNTPGCSCSWPVCTRN | -0.27 | 21 |
|  | vigno 3 | cyclo-GLPLCGETCVGGTCNTPGCSCSWPVCTRN | -0.27 | 21 |
|  | vigno 4 | cyclo-GLPLCGETCVGGTCNTPACSCSWPVCTRN | -0.27 | 21 |
|  | chacur 1 | cyclo-GLPVCGETCVGGTCNTPGCTCSWPICTRN | -0.27 | 17 |
|  | cO22 | cyclo-GLPICGETCVGGTCNTPGCTCSWPVCTRN | -0.27 | 17 |
| Sequences were obtained from the CyBase databank (Wang et al., 2007) and by Hellinger et al. (Hellinger et al., 2015) and net charge and ratio of hydrophilic residues determined using the PeptideCalculator by Bachem (https://www.bachem.com/knowledge-center/peptide-calculator/). | | | | |

**References**

Hellinger, R., Koehbach, J., Soltis, D. E., Carpenter, E. J., Wong, G. K.-S., and Gruber, C. W. (2015). Peptidomics of Circular Cysteine-Rich Plant Peptides: Analysis of the Diversity of Cyclotides from Viola tricolor by Transcriptome and Proteome Mining. *J. Proteome Res.* 14, 4851–4862. doi:10.1021/acs.jproteome.5b00681.

Wang, C. K. L., Kaas, Q., Chiche, L., and Craik, D. J. (2007). CyBase: a database of cyclic protein sequences and structures, with applications in protein discovery and engineering. *Nucleic Acids Res.* 36, D206–D210. doi:10.1093/nar/gkm953.
